# Complete Luxation of the Lower Jaw Reduced on the Eighty Seventh Day

**Published:** 1858-01

**Authors:** 


### ARTICLE IX.

*Complete Luxation of the Lower Jaw reduced on the eighty-seventh day.* Translated from the French for this Journal.

OLD luxations of the lower jaw have always attracted the attention of surgeons, and the opinions as to the proper treatment have been various. The most prudent left the patients to themselves, believing that in time the lower jaw would rise sufficiently to come in contact with the upper, and so still render possible the movements of mastication. Others more bold, wishing to bring about reduction, had recourse to powerful measures, such as the wooden wedges of Guillaume de Salicet, the sling with the addition of the packing stick, the levers, the pincers of Junk, Atti & Stromeyer. But it has been generally agreed to discard from the beginning the most simple processes as being necessarily inefficient.

The depression of the jaw by simple pressure with the fingers, presents an incontestable advantage, for this pressure allows the surgeon to take an exact account of what happens during the effects at reduction, and to measure the force to be employed to overcome the resistance. This force, it is true, is often insufficient, and then we may have recourse to more energetic measures; but sometimes, also, it is sufficient, and we obtain, without great trouble, an almost unhoped for result.

The following case for the communication of which we are indebted to M. Charnal, a distinguished *interne* of the hospital, appears to us sufficiently remarkable to be reported.

On the 16th of May, 1857, Madame F——, 64 years of age, landed proprietress at E—— (Seine et Marne,) moving rapidly across a room in which were deposited some farming utensils, caught her foot in a cord and fell forward. The fall was so violent that the lady immediately lost consciousness. When she was picked up, a large wound of the head in the left temporo-frontal region, and a notable deformity of the features were observed.

For eight days, the physician in charge had to struggle against grave cerebral symptoms, which finally yielded. Some days later, the patient being better, wished to take some solid food, but found it impossible to approximate her jaws. The physician consulted upon that subject said that it was nothing, and that she must not concern herself about it.

On the 6th of August, Madame F—— finding no change in her situation, determined to come to Paris, and entered the Municipal *Maison de Santé.*

At the first glance it is easy to recognize a luxation of both condyles of the lower jaw. The chin thrust forwards and downwards, the impossibility of bringing the two jaws into contact, the difficulty of pronunciation, the peculiar deformity of the features are sufficient signs. The lips uniting so as completely to close the mouth, there is no trickling of saliva.

The lower jaw has not completely lost its mobility, and the patient can, at will, augment the separation of the dental arches. This separation is at the minimum two centimetres, (about .8 of inch,) and at the maximum three centimetres; besides, at the level of the incisors the lower is found to be nearly one centimetre in front of the upper. Finally, the fingers introduced into the mouth detect the coronoid apophyses of the lower maxillary engaged under the malar bone.

The patient having no recollection of what happened at the time of the accident, it is impossible for us to indicate by what mechanical force the luxation was produced.

Upon her admission, i. e., the 83d day after the accident, M. Demarquay attempted to reduce the luxation with the thumbs upon the molar teeth, so as to depress the whole jaw, (Nelaton's process.) This first attempt was unsuccessful. The patient was subjected to chloroform vapor, and when muscular relaxation was produced, M. Demarquay made another attempt. Under the influence of strong pressure, some crackling was heard, the jaw descended a little; then, on carrying it backward, it became evident that the right condyle had re-entered its glenoid cavity. M. Demarquay then directed his efforts to the left side; but immediately the luxation was reproduced upon the right, but reduced again with great facility. The jaw was then fixed in this position by the aid of a *funda.*

This first sitting lasted twenty minutes. The patient remained in this condition for four days without any reproduction of the luxation on the right side, and without the smallest accident.

Finally, on the 10th of August, the 87th day after the accident, M. Demarquay having taken the precaution to supply himself with Stromeyer's pincers, makes a new attempt by pressing with the thumb upon the left molars; soon a tolerably loud crackling is heard, the jaw descends, it is rapidly carried backwards and the reduction is complete. Immediately the patient is able to separate and reclose the dental arches without the least difficulty. The handkerchief is again fixed by a funda. This second sitting lasted ten minutes.

On the 11th it is clear that the motions of elevation and depression are made with facility; but is also evident that the place of the lower dental arch is a little anterior to that of the upper, a phenomenon almost constantly present after old luxations, and which depends upon the filling up of the glenoid cavities with deposits of lymph or with cellular tissue, so that the condyles cannot immediately resume their normal position.

Finally, on the 14th of August, the patient can, without inconvenience, exercise the motions of mastication and leaves the house.

This fact may be placed alongside of that of Donovan, who reduced a luxation solely by the pressure of the thumb, with great force it is true, on the 98th day. In both cases the luxation was first reduced on one side, second condyle not being reduced till after several day's rest.

This case shows us, that in luxations of the lower jaw, even three months old.

1. We must not give up all hope of reduction and leave the patient to himself.

2. Before employing violent means, we should always use the simpler processes, which often give unexpected results.

3. The process of M. Nelaton may be sometimes successfully employed.

4. Finally, the successive reduction of the condyles, as Monteggia & Hey advise, seems to diminish considerably the resisting force.—*Gazette des Hôpitaux, Aug. 22d,* 1857.

